# Neuropsychiatric risk in children with intellectual disability of genetic origin: IMAGINE, a UK national cohort study

**DOI:** 10.1016/S2215-0366(22)00207-3

**Published:** 2022-09

**Authors:** Jeanne Wolstencroft, Francesca Wicks, Ramya Srinivasan, Sarah Wynn, Tamsin Ford, Kate Baker, Samuel J R A Chawner, Jeremy Hall, Marianne B M van den Bree, Michael J Owen, IMAGINE Study, Jeanne Wolstencroft, Francesca Wicks, Ramya Srinivasan, Marie Erwood, Amy Lafont, Husne Timur, Zheng Ye, Susan Walker, Frida Printzlau, Manoj Juj, Sarah Davies, Hayley Denyer, Alice Watkins, Eleanor Kerry, Nadia Coscini, Nasrtullah Fatih, Anna Lucock, Spiros Denaxas, William Mandy, Neil Walker, Sarah Wallwork, Eleanor Dewhurst, Andrew Cuthbert, Aimee Challenger, Sophie Andrews, Peter Holmans, Samantha Bowen, Karen Bradley, Philippa Birch, Molly Tong, Nicola Lewis, Sinead Ray, Matthew Sopp, Hayley Moss, Sarah Wynn, Beverley Searle, Lisa Robertson, Jonathan Berg, Anne Lampe, Shelagh Joss, Paul Brennan, Alison Kraus, Nayana Lahiri, Astrid Weber, Myfanwy Rawson, Diana Johnson, Pradeep Vasudevan, Rachel Harrison, Denise Williams, Eamonn Maher, Usha Kini, Fleur Van Dijk, Virginia Clowes, Jana Gurasashvilli, Sahar Mansour, Muriel Holder-Espinasse, Amy Watford, Julia Rankin, Diana Baralle, Annie Procter, Tamsin Ford, Kate Baker, Samuel Chawner, Jeremy Hall, Marianne B M Van den Bree, Michael J Owen, David Skuse, F Lucy Raymond, David Skuse, F Lucy Raymond

**Affiliations:** a NIHR BRC Great Ormond Street Institute of Child Health, University College London, London, UK; b School of Clinical Medicine, University of Cambridge, Cambridge, UK; c Cambridge University Hospitals NHS Foundation Trust, Cambridge Biomedical Campus, Cambridge, UK; d NIHR Bioresource, Cambridge Biomedical Campus, Cambridge, UK; e Medical Research Council Centre for Neuropsychiatric Genetics and Genomics, Division of Psychological Medicine and Clinical Neurosciences, Cardiff University, Cardiff, UK; f Neuroscience and Mental Health Research Institute, Cardiff University, Cardiff, UK; g Cardiff University Centre for Human Developmental Science, School of Psychology, Cardiff University, Cardiff, UK; h UNIQUE Charity, Oxted, UK; i UCL Division of Psychiatry, University College London, London, UK; j MRC Cognition and Brain Sciences Unit, University of Cambridge, Cambridge, UK

## Abstract

**Background:**

Children with intellectual disability frequently have multiple co-morbid neuropsychiatric conditions and poor physical health. Genomic testing is increasingly recommended as a first-line investigation for these children. We aim to determine the effect of genomics, inheritance, and socioeconomic deprivation on neuropsychiatric risk in children with intellectual disability of genetic origin as compared with the general population.

**Methods:**

IMAGINE is a prospective cohort study using online mental health and medical assessments in a cohort of 3407 UK participants with intellectual disability and pathogenic genomic variants as identified by the UK's National Health Service (NHS). Our study is on a subset of these participants, including all children aged 4–19 years. We collected diagnostic genomic reports from NHS records and asked primary caregivers to provide an assessment of their child using the Development and Well-Being Assessment (DAWBA), the Strengths and Difficulties Questionnaire (SDQ), the Adaptive Behaviour Assessment System 3 (ABAS-3), and a medical history questionnaire. Each child was assigned a rank based on their postcode using the index of multiple deprivation (IMD). We compared the IMAGINE cohort with the 2017 National Survey of Children's Mental Health in England. The main outcomes of interest were mental health and neurodevelopment according to the DAWBA and SDQ.

**Findings:**

We recruited 2770 children from the IMAGINE study between Oct 1, 2014 and June 30, 2019, of whom 2397 (86·5%) had a basic assessment of their mental health completed by their families and 1277 (46·1%) completed a medical history questionnaire. The mean age of participants was 9·2 years (SD 3·9); 1339 (55·9%) were boys and 1058 (44·1%) were girls. 355 (27·8%) of 1277 reported a seizure disorder and 814 (63·7%) reported movement or co-ordination problems. 1771 (73·9%) of 2397 participants had a pathogenic copy number variant (CNV) and 626 (26·1%) had a pathogenic single nucleotide variant (SNV). Participants were representative of the socioeconomic spectrum of the UK general population. The relative risk (RR) of co-occurring neuropsychiatric diagnoses, compared with the English national population, was high: autism spectrum disorder RR 29·2 (95% CI 23·9–36·5), ADHD RR 13·5 (95% CI 11·1–16·3). In children with a CNV, those with a familial variant tended to live in more socioeconomically deprived areas than those with a de novo variant. Both inheritance and socioeconomic deprivation contributed to neuropsychiatric risk in those with a CNV.

**Interpretation:**

Children with genomic variants and intellectual disability are at an increased risk of neuropsychiatric difficulties. CNV variant inheritance and socioeconomic deprivation also contribute to the risk. Early genomic investigations of children with intellectual disability could facilitate the identification of the most vulnerable children. Additionally, harnessing parental expertise using online DAWBA assessments could rapidly identify children with exceptional needs to child mental health services.

**Funding:**

UK Medical Research Council and Medical Research Foundation.

## Introduction

The genomic basis of intellectual disability is being unveiled at pace. Large-scale identification of highly penetrant variants that cause developmental delay, intellectual disability, and autism has been achieved using next generation sequencing methods with a trio-based design (parents and child).^1^, ^2^, ^3^ Best practice guidelines recommend exome or genome sequence analysis as a first or second tier investigation for all children presenting with developmental delay or intellectual disability.^4^ Yet, with a few exceptions, the confidence with which a rare genomic variant can be regarded as pathogenic is not matched by an equivalent confidence about the implications of that finding for the child's future neuropsychiatric profile. Compared with the general population, children with intellectual disability have substantial additional needs in terms of physical and mental health, but we know little about the influence of most identified rare genomic variants on the long-term outcome of a child with intellectual disability.^5^ Most previous studies of children with intellectual disability are limited by modest sample sizes^5^, ^6^ or have selected participants from large epidemiological cohorts in which the proportion of children with moderate to profound intellectual disability was small.^7^, ^8^ So far, no national cohort study of intellectual disability has collected genomic data at scale. We do not know to what extent prognosis is influenced by environmental factors, such as socioeconomic deprivation, or genetic factors, such as the inheritance of the genomic variant (familial or de novo). This study was designed to assess social and demographic influences on the physical and mental health of a national cohort of children and young people with rare genomic disorders associated with intellectual disability. The study was also designed to make a comparison of prevalence of neuropsychiatric diagnoses with the equivalent diagnostic data provided by the UK National Survey of Children's Mental Health 2017.^9^ If physical and mental health care needs can be predicted at the point of genetic diagnosis, then early personalised interventions could benefit the most vulnerable children.

**Research in context**

**Evidence before this study**

Previous studies of neuropsychiatric risk in children with intellectual disability have either used small-scale cohorts or were not designed to evaluate a wide range of mental health issues. Studies that have sought evidence for genetic predisposition have, in almost all instances, started from a phenotype of interest (such as autism spectrum disorder) and then screened for pathogenic variants. We searched titles and abstracts in PubMed for publications in English from database inception until June 11, 2021, using the search terms ((child*) AND ((developmental delay) OR (intellectual disability)) AND (mental health) AND (cohort). Only one national cohort survey of children's mental health has reported on the increased risk of mental health and neurodevelopmental disorders among children with intellectual disability using standardised measures. Other relevant cohort studies have focused on the identification of specific neurodevelopmental disorders in a population (eg, autism spectrum disorder and ADHD) that might incidentally be associated with intellectual disability, but have not reported on co-occurring behavioural or emotional problems. One genotype-first study of developmental delay in non-syndromic children has been published, but this did not systematically evaluate neurodevelopmental risk or mental health.

**Added value of this study**

Our nationally representative cohort of children aged 4–19 years had identified pathogenic genomic variants encompassing copy number variants and single nucleotidevariants that are more varied than any previous genotype-first investigation of neurodevelopmental risk. Data were collected using standardised measures of child mental health that are equivalent to those used in UK national surveys and thus allow direct comparison with general population data collected contemporaneously. The unique contribution of this investigation is that it provides evidence from a genotype-first investigation of neuropsychiatric risk, with the predisposing genomic variants reported by a UK National Health Service diagnostic protocol. The addition of data on socioeconomic status is based on a multifaceted UK index of multiple deprivation; a variable that has not previously been used in epidemiological studies of mental health risk in children with developmental delay.

**Implications of all the available evidence**

Routine genomic testing is identifying pathogenic variants in an increasing proportion of children with developmental delay but, except for a few relatively well-studied variants, the implications of a genomic disorder for a child's future mental health is currently unknown. Intellectual disabilities are generally associated with an increased risk of neurodevelopmental disorders, as well as emotional and behavioural problems, but this study has shown that the risk is amplified considerably in children whose developmental delay has an identifiable genetic cause. Our findings have implications for the clinical management of such children and indicate an urgent need for early assessment and intervention.

## Methods

### Study design and participants

The Intellectual Disability and Mental Health: Assessing the Genomic Impact on Neurodevelopment (IMAGINE) study is a cohort study of 3407 UK participants who were recruited between Oct 1, 2014, and June 30, 2019. To be eligible, participants were required to be aged at least 4 years at the time of enrolment, to have developmental delay or an intellectual disability diagnosis made by a clinical care team, and to have a confirmed molecular genetic diagnosis documented from an accredited diagnostic laboratory. Pathogenic variants were classified according to the American College of Medical Genetics and Genomics guidelines and only those participants with pathogenic or likely pathogenic variants were included.^10^ Recruitment to the study was by referral from all UK regional genetics centres (2596 [76·2%] of 3407) and self-referrals or patient support groups, such as UNIQUE Charity (811 [23·8%] of 3407). This study is focused on a subset of 2570 (81·3% of 3407) individuals who were aged 4–19 years. A parent or guardian provided consent on behalf of children younger than 16 years. All participating children received a copy of a storybook, *Avery,* written for this study to facilitate a discussion between parents and children about research involvement.^11^ For individuals older than 16 years who did not have capacity, consultees acted on their behalf. This study was approved by London Queen Square Research Ethics Committee (14/LO/1069).

### Procedures

We obtained diagnostic genomic reports, including genetic inheritance information (de novo or inherited) if available, from participants' UK National Health Service (NHS) medical records or directly from their families (appendix p 2). For individuals with multiple genetic variants, subsequent data analysis was based on the most pathogenic variant.

Primary caregivers were invited to complete all questionnaires regarding their child, including online assessments of their child's educational progress and physical and mental health using the Development and Well-Being Assessment (DAWBA), which is organised into modules and includes a combination of open text, binary questions, and Likert scale questions, and the Strengths and Difficulties Questionnaire (SDQ; appendix p 3).^12^, ^13^Daily living skills were measured using the Adaptive Behaviour Assessment System 3 (ABAS-3).^14^ A developmental quotient was calculated from primary caregivers' estimates of the child's mental age divided by their chronological age.^7^, ^15^ A structured supplemental medical history questionnaire gathered information about the child's time in utero, birth, early development, current medical problems, medication, and included information on special educational needs, educational health care plans^16^ and disability living allowance.^17^ Postcodes of participating family homes were ranked on an index of multiple deprivation (IMD), provided by the UK Office for National Statistics.^18^, ^19^ In the current English Indices of Deprivation 2019, seven domains of deprivation are considered and weighted as follows: income (22·5%), employment (22·5%), education (13·5%), health (13·5%), crime (9·3%), barriers to housing and services (9·3%), and living environment (9·3%). The indices of multiple deprivation for Wales, Scotland, England, and Northern Ireland are calculated separately.

The Mental Health of Children and Young People in England 2017 Survey is funded by the Department of Health and Social Care, commissioned by NHS Digital, and carried out by the National Centre for Social Research, the Office for National Statistics, and Youthinmind, to provide data on trends in child mental health in England. DSM-5 disorder prevalence rates from the 2017 national survey were used as a comparator group in this study.^9^

### Outcomes

The main outcomes were mental health and neurodevelopment according to the DAWBA and SDQ, and daily living skills according to the ABAS-3.

### Choice of mental health outcomes

We chose the DAWBA and SDQ as our mental health outcome measures as both have been used in national studies of children's mental health in the UK and international surveys of child psychopathology.^20^, ^21^, ^22^ The measures are available in over 20 languages. The DAWBA is a comprehensive psychiatric interview that provides DSM-5-compatible diagnoses and broader measures of adjustment and family functioning. DAWBA makes a clear distinction between problem behaviour in general and specific psychiatric disorders, which is important in the intellectual disability population.^7^, ^23^, ^24^

To maximise validity and reliability, we used rating procedures identical to those used in the Mental Health of Children and Young People in England 2017 Survey.^9^ Diagnoses, using DSM-5 criteria, were assigned by two independent experienced clinicians.^25^ Inter-rater reliability was checked by co-rating 147 randomly chosen participants with the team that did the National Survey of Children's Mental Health,^9^ and all kappa values for diagnostic categories were more than 0·7 (appendix p 3).

The SDQ assesses children's emotional and behavioural adjustment in dimensional terms.^8^, ^23^, ^26^, ^27^ The SDQ has been validated for children with intellectual and developmental disabilities.^23^ The SDQ includes five scales that measure: emotional symptoms; conduct problems; hyperactivity, impulsivity, and inattention difficulties; peer relationship problems; and prosocial behaviour. The first four of these scales are combined to create a total difficulties score. High scores are indicative of greater mental health difficulty and scores above the 90th percentile (or ≥17) indicate a high probability of a diagnosable psychiatric disorder.^27^

### Statistical analysis

We did four sets of analyses. First, we computed descriptive statistics to describe the cohort's characteristics in the following domains: genetics; development, education, and adaptive impairment; socioeconomic status; and neuropsychiatric risk. Secondly, we did group comparisons using χ^2^ tests on the prevalence of DAWBA diagnoses between the IMAGINE cohort and the UK national survey. Then, we did the third and fourth set of analyses on a subset of the cohort who had a copy number variant (CNV) of known inheritance (ie, de novo or familial status). The third analysis compared the behavioural phenotypes and neuropsychiatric risk of children on the basis of the inheritance of their CNV. The Bonferroni method was used to adjust the threshold of significance for multiple comparisons in the second and third sets of analyses. Our fourth and final set of analyses investigated the association between variants: inheritance (de novo or familial status), IMD quintile, and SDQ, using multivariable hierarchical linear regressions. Model 1 predicted the degree of behaviour difficulties (SDQ total score) from the IMD quintile and variant inheritance. Model 2 adjusted for confounding factors including sex, age at diagnosis, developmental quotient, and physical health problems. Model 3 added an interaction factor (deprivation × inheritance). All data were analysed in SPSS version 24.

### Role of the funding source

The study funders and sponsors were not involved in the study design, the collection, analysis, and interpretation of data, in the writing of the report, or in the decision to submit the paper for publication.

## Results

**Figure 1:**
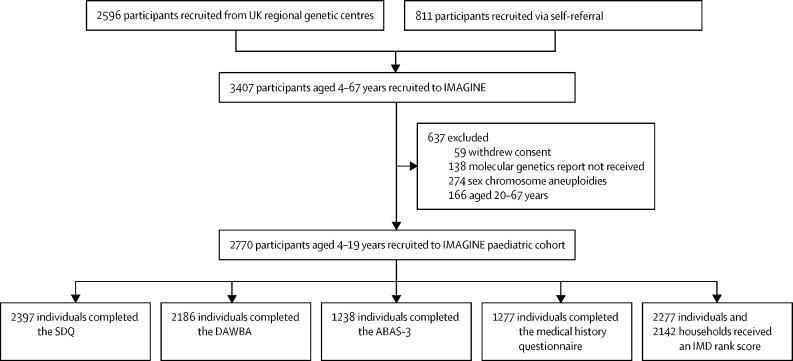
Cohort flow chart SDQ=Strengths and Difficulties Questionnaire. DAWBA=Development and Well-Being Assessment. ABAS-3=Adaptive Behaviour Assessment System 3. IMD=index of multiple deprivation.

We recruited a total of 3407 participants to the IMAGINE study between Oct 1, 2014 and June 30, 2019 (figure 1). The main recruitment source was UK Regional Genetic Centres (2596 [76·2%] people) and the remaining 811 (23·8%) people were recruited through self-referral. 2770 (81·3%) of 3407 participants were aged 4–19 years, of whom 2397 (86·5%) had a basic assessment of their mental health completed by their families (figure 1; appendix p 29). The mean age of this subsample was 9·2 years (SD 3·9), and 1339 (55·9%) of 2397 participants were boys and 1058 (44·1%) were girls (table 1). Ethnicity data were not collected.

**Table 1:** Cohort characteristics

	**Participants (n=2397)**
**Variant type**	
Copy number variant	1771 (73·9%)
Single nucleotide variant	626 (26·1%)
**Variant inheritance**	
Familial	645 (26·9%)
de novo	940 (39·2%)
Not determined	812 (33·9%)
**Sex**	
Male	1339 (55·9%)
Female	1058 (44·1%)
**Age, years**	
4–8	1211 (50·5%)
9–11	531 (22·2%)
12–16	533 (22·2%)
17–19	122 (5·1%)
**Age at diagnosis, years**	
<4	710/2349 (30·2%)
4–8	1045/2349 (44·5%)
9–11	341/2349 (14·5%)
12–16	227/2349 (9·7%)
17–19	26/2349 (1·1%)
IMD quintile by household*	
1	431/2142 (20·1%)
2	406/2142 (19·0%)
3	407/2142 (19·0%)
4	427/2142 (19·9%)
5	471/2142 (22·0%)
**Mean intellectual functioning**†	
Developmental quotient‡	0·55 (0·2)
Mental age, years	4·94 (3·0)
**General physical health**§	
Very good	635/2297 (27·6%)
Good	944/2297 (41·1%)
Fair	567/2297 (24·7%)
Bad	119/2297 (5·2%)
Very bad	32/2297 (1·4%)
**Adaptive Behaviour Assessment System 3**	
Extremely low	817/1238 (66·0%)
Low	238/1238 (19·2%)
Below average	120/1238 (9·7%)
Average	62/1238 (5·0%)
Above average	1/1238 (<0·1%)
**SDQ total score (0–40)**	
Close to average (0–13)	406 (16·9%)
Slightly raised (14–16)	334 (13·9%)
High (17–19)	378 (15·8%)
Very high (20–40)	1279 (53·4%)

Data are n (%) or mean (SD). IMD=index of multiple deprivation. SDQ=Strengths and Difficulties Questionnaire. DAWBA=Development and Well-Being Assessment.

* IMD quintile 1=most deprived, 5=least deprived.

† Mean intellectual functioning data were available from 1911 participants.

‡ The developmental quotient was calculated from primary caregivers' estimates of the child's mental age divided by their chronological age.

§ General physical health was estimated using primary caregiver ratings on the DAWBA (5-point Likert scale from very bad to very good).

1771 (73·9%) of 2397 individuals with SDQ scores available had a pathogenic CNV and 626 (26·1%) had a pathogenic single nucleotide variant (SNV). Familial CNV or SNV variants were identified in 645 (26·9%) individuals; de novo CNV or SNV variants were identified in 940 (39·2%) individuals; and in 812 (33·9%) individuals the parental results were not available to the study, thus, familial or de novo status could not be determined for the pathogenic variant (appendix p 29; table 1; figure 1).

The mean age at diagnosis of a pathogenic CNV was 5·4 years (SD 3·7). In total, 961 different CNV loci were observed within the cohort (appendix p 2). For the 1105 individuals in whom the CNV inheritance was known, 564 (51·0%) had a familial CNV variant compared with 541 (49·0%) who had a de novo CNV variant. The average age at diagnosis of a pathogenic SNV was 7·8 years (SD 4·2). Pathogenic variants in 205 different single genes were observed (appendix p 2). Of the 480 individuals in whom the SNV inheritance was known, 81 (16·9%) variants were documented as familial, compared with 399 (83·1%) that were documented as de novo.

Most children in the cohort had delayed developmental milestones according to primary caregivers' reports; the average age at first walking unsupported was 23·2 months (SD 13·5), and 1735 (72·4%) of 2397 children had delayed language skills. 912 (38·0%) of 2397 children attended specialised education units or schools, 953 (39·8%) attended mainstream school with classroom assistance, 165 (6·9%) attended mainstream school without allocated support, 111 (4·6%) were not at school, and for 256 (10·7%) individuals the type of schooling was not documented. Supplemental medical history information, provided by 1277 individuals' primary caregivers, indicated that 976 (76·4%) had special educational needs or an education health care plan. 978 (76·6%) of the 1277 individuals' caregivers received a disability living allowance for their child. The ABAS-3 was completed for 1238 (44·7%) of 2770 children: 62 (5·0%) scored in the average range, 120 (9·7%) in the below average range, 238 (19·2%) in the low range and 817 (66·0%) in the extremely low range according to ABAS-3 norms (table 1).

**Figure 2:**
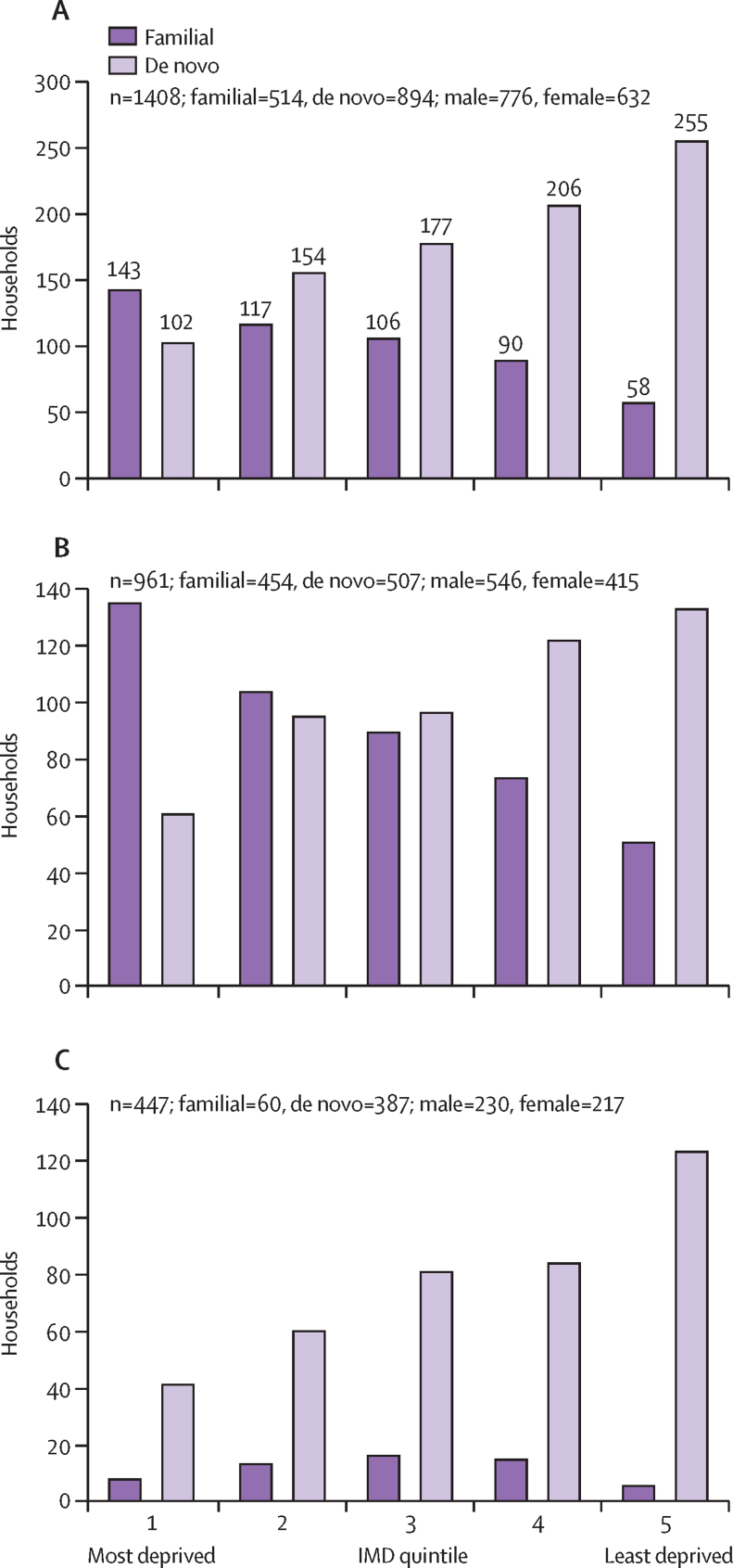
Variant inheritance by IMD quintile for all variants of known inheritance (A), CNV variant inheritance (B), and SNV variant inheritance (C) IMD ranks by UK nations were combined to examine group differences between those households with an inherited and de novo variant. Households were scored once regardless of number of individuals within the household who had genetic variants. IMDs for variants of unknown significance are not represented (n=734). The first quintile includes the most deprived postcodes and the fifth quintile the least deprived postcodes. IMD=index of multiple deprivation. CNV=copy number variant. SNV=single nucleotide variant.

Of the 2397 children for whom measures of mental health were available, residential postcodes linked to IMD ranks were available for 2277 participants from 2142 households. The distribution of IMD ranks approximated a uniform distribution; the cohort was representative of the UK national population based on IMD quintiles (table 1; appendix p 29). Households of children with a familial variant were over-represented in socioeconomically more deprived quintiles (quintiles 1 and 2), and households of children with a de novo variant were over-represented in the least socioeconomically deprived households (quintiles 4 and 5; figure 2).

The SDQ scores (n=2397) revealed a high prevalence of behavioural difficulties compared with the UK national survey norm (table 1). 1992 (83·1%) of 2397 individuals scored above the clinical cutpoint (14 and higher), compared with 20% of the general population of equivalent age and sex. Of these children, 334 (13·9%) of 2397 individuals had slightly raised scores (14–16), 378 (15·8%) had high scores (17–19), and 1279 (53·4%) had very high scores (20–40). Subscale scores were also higher than the UK national average (appendix p 29).

2186 IMAGINE DAWBA diagnoses were compared with the 7654 DAWBA prevalence estimates from the UK 2017 National Survey of Child and Adolescent Mental Health; including diagnoses in 960 (43·9%) girls and 1226 (56·1%) boys in IMAGINE and 3803 (49·7%) girls and 3851 (50·3%) boys in the national survey (table 2). Clinically significant neuropsychiatric disorders were observed in 1161 (53·1%) of the 2186 children with completed DAWBAs, compared with 12·8% (relative risk [RR] 4·1 [95% CI 3·9–4·5]; p<0·0001) in the 2017 national survey. Autism spectrum disorder diagnostic criteria were met in 776 (35·5%) of 2186 individuals compared with 92 (1·2%) of 7654 individuals (RR 29·2 [95% CI 23·9–36·5]; p<0·0001) in the national survey (table 2). ADHD diagnostic criteria were met in 473 (21·6%) of 2186 individuals compared with 123 (1·6%) of 7654 individuals (RR 13·5 [95% CI 11·1–16·3]; p<0·0001; table 2) in the national survey. Oppositional defiant disorders were also relatively common in the IMAGINE sample (264 [12·1%] *vs* 222 [2·9%]; RR 4·2 [95% CI 3·5–5]; p<0·0001), but the rates of conduct disorder were not higher than in the national survey (34 [1·6%] *vs* 130 [1·7%]; RR 0·9 [95% CI 0·6–1·3]; p=0·71). Anxiety disorders were identified in 232 (10·6%) individuals in the IMAGINE study compared with 551 (7·2%) in the national survey (RR 1·5 [95% CI 1·3–1·7]; p<0·0001). Rates of depression were significantly lower in the IMAGINE cohort than in the national survey (9 [0·4%] *vs* 161 [2·1%]; RR 0·2 [95% CI 0·1–0·4]; p<0·0001). Of the 1161 individuals in the IMAGINE cohort who met the criteria for any psychiatric diagnosis, 483 (41·6%) had two or more co-occurring disorders, of which the most frequent co-occurring conditions were autism spectrum disorder and ADHD (247 [21·3%] of 1161 individuals).

**Table 2:** Neurodevelopmental and mental health diagnoses

		IMAGINE (n=2186*)	**2017 UK national survey (n=7654)**	**p value**†	**RR (95% CI)**
Emotional disorders		236 (10·8)	620 (8·1)	<0·0001	1·3 (1·2–1·5)
	Anxiety	232 (10·6)	551 (7·2)	<0·0001	1·5 (1·3–1·7)
	Depression	9 (0·4)	161 (2·1)	<0·0001	0·2 (0·1–0·4)
Behavioural disorders		283 (12·9)	352 (4·6)	<0·0001	2·8 (2·4–3·3)
	Oppositional defiant disorder	264 (12·1)	222 (2·9)	<0·0001	4·2 (3·5–5·0)
	Conduct disorder	34 (1·6)	130 (1·7)	0·71	0·9 (0·6–1·3)
ADHD		473 (21·6)	123 (1·6)	<0·0001	13·5 (11·1–16·3)
Autism spectrum disorder		776 (35·5)	92 (1·2)	<0·0001	29·2 (23·9–36·5)

Data are n (%). Threshold of significance corrected for multiple comparisons using the Bonferroni correction method is p=0·006. RR=relative risk.

* Participants aged 5–19 years.

† Calculated using χ^2^ test of independence.

1277 (46·1%) caregivers completed a supplemental medical history questionnaire (appendix p 30). 1195 (93·6%) reported at least one relevant physical health problem. 355 (27·8%) children had a history of seizures; the most common were absence seizures (148 [41·7%] of 355), generalised tonic-clonic seizures (120 [33·8%]), and febrile seizures (94 [26·5%]; appendix p 30). 188 (53%) of 355 individuals with a history of seizures were on specific anti-epileptic medication. Other physical health problems were common: 825 (64·6%) of 1277 reported disturbed sleep, 814 (63·7%) had motor or movement disorders, 588 (46·0%) had fine motor control problems, and 24 (1·9%) had cerebral palsy (appendix p 30).

Variant inheritance was examined for its contribution to risk of neuropsychiatric disorder in the CNV group (de novo 541, familial 564; table 3). Too few familial SNVs were observed for comparison (de novo 399, familial 81; appendix p 29). Children with a de novo CNV variant were more impaired in their intellectual functioning, but not in their adaptive functioning, than those with a familial variant (table 3). In contrast, more severe behavioural and emotional problems were observed in participants with a familial variant (table 3). Those with a familial CNV variant were also at a higher risk of specific neurodevelopmental diagnoses, including autism spectrum disorder and ADHD, than those with a de novo variant (autism spectrum disorder RR 1·6 [95% CI 1·4–1·9]; p<0·0001 and ADHD RR 1·9 [95% CI 1·5–2·5]; p<0·0001) and those with a familial CNV variant were more likely to live in more deprived socioeconomic areas than those with a familial CNV variant (table 3).

**Table 3:** Copy number variant group participant characteristic comparison by variant inheritance

			**Familial**	**de novo**	**p value**
Mean age, years (n=1106)			8·7 (3·6)	8·9 (3·9)	0·64
Mean age at diagnosis, years (n=1098)			6 (3·6)	4·7 (3·9)	<0·0001
Sex*					0·0002
	Male		356/565 (63%)	281/541 (52%)	
	Female		209/565 (37%)	260/541 (48%)	
IMD quintile by household†					<0·0001
	1		135/454 (30%)	61/411 (12%)	..
	2		104/454 (23%)	95/411 (19%)	..
	3		90/454 (20%)	96/411 (19%)	..
	4		74/454 (16%)	122/411 (24%)	..
	5		51/454 (11%)	133/411 (26%)	..
General physical health†‡					0·99
	Very good		146/549 (27%)	139/522 (27%)	..
	Good		232/549 (42%)	231/522 (44%)	..
	Fair		134/549 (24%)	120/522 (23%)	..
	Bad		32/549 (6%)	27/522 (5%)	..
	Very bad		5/549 (1%)	5/522 (1%)	..
Mean mental age, years (n=855)‡			5·5 (3·0)	4·8 (3·0)	0·0003
Mean developmental quotient (n=855)‡§			0·6 (0·2)	0·5 (0·3)	<0·0001
Mean ABAS-3 (n=549)			66·4 (13·7)	64·2 (13·1)	0·091
Mean total SDQ score (n=1106)			22·7 (6·5)	18·5 (6·5)	<0·0001
DAWBA (n=1021)*					
	Emotional disorders		78/529 (14·7%)	40/492 (8·1%)	0·0010
		Anxiety	77/529 (14·5%)	40/492 (8·1%)	0·0013
		Depression	5/529 (0·9%)	1/492 (0·2%)	0·12
	Behavioural disorders		101/529 (19·1%)	49/492 (10·0%)	<0·0001
		Oppositional defiant disorder	96/529 (18·1%)	48/492 (9·8%)	0·0001
		Conduct disorder	13/529 (2·5%)	4/492 (0·8%)	0·040
	Hyperactivity disorder		145/529 (27·4%)	69/492 (14%)	<0·0001
	Autism spectrum disorder		242/529 (45·7%)	141/492 (28·7%)	<0·0001

Data are n (%) or mean (SD). Threshold of significance corrected for multiple comparisons using the Bonferroni correction method α=0·002. General physical health was estimated using primary caregivers' ratings on the DAWBA (5 point Likert scale from very bad to very good). IMD quintile 1=most deprived, 5=least deprived. See appendix (p 29) for summary of n numbers. IMD=index of multiple deprivation. ABAS-3=Adaptive Behaviour Assessment System 3. SDQ=Strengths and Difficulties Questionnaire. DAWBA=Development and Well-Being Assessment.

* Calculated using χ^2^ test of independence.

† Calculated using two-sample Kolmogorov–Smirnov test.

‡ DAWBA skip rules affect number of responses.

§ The developmental quotient was calculated from primary caregivers' estimates of the child's mental age divided by their chronological age (0=low developmental level, 1=high developmental level).

There was a higher proportion of boys among those with familial variants than in the overall cohort (table 3): 356 (63·0%) boys and 209 (37·0%) girls with familial genomic variants. In children with a CNV, there was a greater severity of behavioural and emotional disorders in those whose variant was familial than in those whose variant was de novo, and there was an association with socioeconomic deprivation. From the hierarchical multivariable linear regressions that tested the significance of these associations, model 1 showed greater socioeconomic deprivation and possession of a familial variant both contributed to behavioural difficulties (B_IMD_=–0·48, SE=0·16, p=0·003; B_inheritance_=4·03, SE=0·46, p<0·0001). Model 2 adjusted for confounders including sex, age of diagnosis, developmental quotient, and physical health (table 4); inheritance and the degree of deprivation remained predictors of behavioural difficulties (B_IMD_=–0·34, SE=0·16, p=0·033; B_inheritance_=3·7, SE=1·16, p<0·0001). In model 3, no significant interaction was found between the index of multiple deprivation and inheritance of the genomic variant (p=0·41; table 4).

**Table 4:** Association between SDQ and IMD quintile by variant inheritance in CNV group (n=806)

	**Model 1**			**Model 2**			**Model 3**		
	B (SE)	Standardised β	p	B (SE)	Standardised β	p	B (SE)	Standardised β	p
IMD	−0·48 (0·16)	−0·10	0·0030	−0·34 (0·16)	−0·07	0·033	−0·47 (0·23)	−0·10	0·036
de novo or familial	4·03 (0·46)	0·31	<0·0001	3·67 (0·46)	0·28	<0·0001	2·90 (1·06)	0·22	0·0062
inheritance × IMD	..	..	..	..	..	..	0·26 (0·32)	0·06	0·41

*R*^2^=0·12, F(2, 803)=56·70, p<0·0001) for model 1; *R*^2^=0·15, F(6, 799)=24·45, p <0·0001) for model 2; *R*^2^=0·16, F(7, 798)=21.04, p<0.0001 for model 3. Model 1 test the associations between SDQ and IMD quintile for individuals by inheritance. Model 2 tests model 1 including confounding variables (sex and developmental level) as indexed by the developmental quotient (developmental age divided by chronological age), age of diagnosis, and physical health (5-point Likert scale from very bad to very good) by primary caregiver's report. Model 3 tests model 2 including an interaction factor (inheritance × IMD). IMD=index of multiple deprivation. SDQ=Strengths and Difficulties Questionnaire. CNV=copy number variant.

## Discussion

Our study, which enrolled over 2700 children, highlighted that intellectual disability of identifiable genetic cause is strongly associated with neurodevelopmental and mental health conditions, and that the risk is higher in those whose genetic condition is inherited than in those in whom the genetic variant is only present in the child, even after adjusting for developmental level, sex, and socioeconomic deprivation.

Our unique approach to measurement of different conditions allowed us to include the assessment of conditions that are typically not included in studies of genetic risk in childhood. Previous studies have either focused almost entirely on the physical consequences of genetic changes,^2^ or they have taken a relatively homogeneous population with a specific neurodevelopmental disorder (such as autism) and sought evidence of specific genomic variants that could have had a causal role.^28^ Although we found that neurodevelopmental conditions were particularly frequently associated with intellectual disability of genetic origin, we discovered that anxiety and oppositional defiant behaviour were also associated. Previous studies that have examined the effect of pathogenic CNVs on child mental health have been small scale, focused on specific neurodevelopmental disorders (such as autism or schizophrenia), and considered only a narrow range of genomic variants. The IMAGINE study comprised a far wider range of CNVs and SNVs, and a greater breadth of neuropsychiatric phenotypes, than any previous investigation of its type.

Consistent with previous work on intellectual disability in populations of children,^7^ we found an association between the degree of children's emotional and behavioural disturbance and families living in socioeconomic deprivation.^29^ Our first novel discovery was that such disorders were more prevalent among children whose genetic condition was inherited than in those in whom it was de novo. The measurable effect of heritable variants on associated risk was largely confined to CNVs because SNVs were usually de novo in origin. Individuals with SNVs were also disproportionately drawn from less socially disadvantaged families than individuals with CNVs, who were identified in a socioeconomically representative cohort.

Considering the important finding that children with inherited CNVs are at increased risk of neuropsychiatric disorders, it is feasible that some parents also might have a degree of cognitive impairment themselves, associated with their carrier status, and be at social and educational disadvantage.^30^ This hypothesis could explain the observation that such families live in conditions of greater multiple deprivation than the general population and would partially explain the association between inherited CNVs and non-specific emotional and behavioural problems.^31^ However, we also found that the neurodevelopmental disorders, ADHD and autism spectrum disorder, were nearly twice as prevalent among children whose CNV was inherited. This finding could reflect some factors that influenced the pathogenicity of the associated CNV, polygenic risk that was also inherited,^32^ or unmeasured environmental factors that the study did not capture. Consistent with previously published data, we found a relative paucity of girls with familial variants compared with boys, supporting the theory of the neuroprotective effect of the female sex.^33^ We found that children with intellectual disability of genetic origins are not only at high risk of mental health and neurodevelopmental disorders, but approximately 30% had a seizure disorder and other complex physical health needs. The children with seizures or absences were not confined to those with genomic variants within known epilepsy genes or genomic loci, but had a wider range of genomic disorders than anticipated, suggesting that the presence of a seizure disorder is a more generalised phenomenon in children with intellectual disability than previously thought.^34^

Our study has some limitations. Recruitment was almost exclusively based on referrals initiated by UK NHS regional genetics centres. Families with a child in whom a pathogenic variant had been diagnosed were approached with information about the IMAGINE study by these centres, and the number of families that declined to take part is unknown. Initial genetic investigations in most children were due to developmental delay. Genetic testing due to suspected autism cannot be excluded, although autism alone is not an indication for genetic investigations according to current NHS guidelines. All participants in the UK came through NHS testing routes, and a diverse range of technologies were used to make genomic diagnoses. The high number of children with CNV reflects historic limitations in diagnostic technologies. The inheritance of each variant was only identifiable in 64% of participants. The study did not include children with intellectual disability without a molecular diagnosis. It is unlikely that these children will have significantly different mental health needs than those with a genetic diagnosis, but our study could not inform this assumption.

Assessments of mental health were mostly obtained online and were based on parental or primary carer's report. It is possible that parents who have a rare genetic disorder themselves, or who are living in socioeconomically disadvantaged circumstances, rated behaviour differently to those with no underlying rare genetic disorders or living in less deprived circumstances. To mitigate against parental bias in reporting (eg, cognitive function), multiple validated and independent assessment tools were used throughout. Additionally, the threshold for referral to services, and difficulties in navigating access to services including genetic testing, could be higher for children with a familial CNV, which would bias the sample to more severely impaired neurodevelopment in children with familial CNVs than children with a de novo CNV. Participation rates in families who volunteered to join the cohort were very high: 85% completed at least one assessment. A strength of our design was that we measured and assessed mental health and neurodevelopmental disorders using the same instruments used in other UK national studies, allowing direct comparisons with general population data. Our diagnostic evaluations were shown to be consistent with the diagnostic decision making of the latest national UK survey of children's mental health. A further strength was that participants were recruited from the NHS genetic service, which is free at the point of delivery and thus demographically and socioeconomically unbiased, and which provided consistent quality of diagnoses, based on accredited diagnostic reports.

Future research should evaluate the emergence of new mental health outcomes over time and investigate sex-differences in these trajectories. The median age of participating children was 9 years at our initial assessment, but serious mental health disorders that are associated with many of the genetic disorders we surveyed do not appear until adolescence or early adult life. We are now following up the families 5 years after our initial evaluation to understand the effect of their genetic disorder on specific educational needs, and to plan for appropriate medical management. At the point of a genetic diagnosis of most of the conditions we identified, often in early childhood, there is little information on the long term mental health and neuropsychiatric needs of these children.

To conclude, our study is the largest survey yet of rare genomic variants that are associated with intellectual disability. The identification of a pathogenic CNV or SNV in a child with developmental delay indicates an exceptionally high risk of the child developing an associated neurodevelopmental disorder or other mental health condition, irrespective of the specific rare genomic variant. Those in whom a genomic variant is inherited are particularly vulnerable. This information should be used to plan targeted assessments and interventions to support families at the earliest opportunity. We would recommend better training for health care providers about the wider use and utility of genetic testing and its value in terms of predicting potential mental health needs of children. We would also recommend better use of parental expertise in pre-assessment of children's needs. Wider use of online assessments of children (eg, DAWBA) could have a substantial impact on rapidly identifying the children in most need of child mental health services, of which there is currently scarce availability in the UK.

For more information on the **IMAGINE study** see https://imagine-id.org/

For more on the **2019 English Indices of Deprivation** please see https://www.gov.uk/government/statistics/english-indices-of-deprivation-2019

For the **UK Data Archive** see https://beta.ukdataservice.ac.uk/datacatalogue/studies/study?id=8621

For the **UNIQUE charity** please see https://rarechromo.org

For more on **DAWBA** see https://www.dawba.info

For the **IMAGINE data access committee** see https://imagine-id.org/healthcare-professionals/datasharing

## Data sharing

The full phenotypic IMAGINE dataset is available from the UK Data Archive under special licence access (SN 8621). Requests for genotype or linked genotypic-phenotypic data can be made through the IMAGINE data access committee.

## Declaration of interests

FLR and ERM were supported by Cambridge National Institute of Health Research (NIHR) Biomedical Research Centre. SJRAC is supported by an Institutional Strategic Support Fund Wellcome Trust Fellowship (204824/Z/16/Z) and RS from a Wellcome Trust grant (211163/Z/18/Z). EM and FLR receive salary support through the University of Cambridge from the NHS in the East of England through the Clinical Academic Reserve. All other authors declare no competing interests.
