# Supplementary material for: Neuropsychiatric risk in children with intellectual disability of genetic origin: IMAGINE, a UK national cohort study
Source: Lancet Psychiatry. 2022 Sep;9(9):715–24. doi: 10.1016/S2215-0366(22)00207-3 (PMC9636306; doi:10.1016/S2215-0366(22)00207-3)
Supplement: Supplementary appendix [file mmc1.pdf]

THE LANCET
Psychiatry

Supplementary appendix

This appendix formed part of the original submission and has been peer reviewed. We post it as supplied by the authors.

Supplement to: Wolstencroft J, Wicks F, Srinivasan R, et al. Neuropsychiatric risk in children with intellectual disability of genetic origin: IMAGINE, a UK national cohort study. *Lancet Psychiatry* 2022; published online August 3. https://doi.org/10.1016/S2215-0366(22)00207-3.

Appendices

Appendix 1: Genetic data

Appendix 2: Supplemental method

Appendix 3: IMAGINE Supplemental Medical History Questionnaire

Appendix 4: Supplementary Tables and Figures

Appendix 5: Data accessibility statement and consortium members

Appendix 6: STROBE reporting guidelines

Appendix 1: Genetic data

Table 1: Example of IMAGINE genetic database available for data sharing upon request

IMAGINE STUDY NUMBER	VARIANT CATEGORY (A= MOST PATHOGENIC)	TYPE OF VARIANT	KARYOTYPE	CHROMOSOME	START	END	BROWSER FORMAT	BAND KARYOTYPE	GENE CONTENT	SIZE (bp)	CONSEQUENCE	TRANSCRIPT ID	RefSeq	CODING SEQUENCE POSITION (c.)	REFERENCE ALLELE	ALTERNATE ALLELE	VARIANT PROTEIN POSITION (p.)	VARIANT AMINO ACID	GENOTYPE	GENETIC TEST USED	FISH PROBE	INHERITANCE	BUILD TYPE
EG1	A	CNV	46,XY	1	1	10412593	chr1:1-10412593	chr1p36.33-p36.22		10412593	Deletion									Array CGH		De Novo	GRCh36/hg18
EG2	A	CNV	46,XY	2	50775890	50947729	chr2:50775890-50947729	chr2p16.3		171840	Deletion									Array CGH		Maternal	GRCh37/hg19
EG3	A	CNV	46,XX	3	166052427	175222778	chr3:166052427-175222778	chr3q26.1-q26.31		9170352	Duplication									Array CGH		Paternal	GRCh36/hg18
EG4	A	SNV		5	176718986	176718986	chr5:176718986-176718986	chr5q35.3	NSD1		Frameshift	ENST00000439151	NM_022455	6290	A	AA	2097	K>KfsX14	Heterozygous	Single gene test		Not determined	GRCh37/hg19
EG5	A	SNV		6	33400030	33400030	chr6:33400030-33400030	chr6p21.32	SYNGAP1		Splice site	ENST00000646630	NM_006772	387+1	G	C			Heterozygous	Whole Exome Sequence		De Novo	GRCh37/hg19
EG6	A	CNV	46,XY	9	753255	4718710	chr9:753255-4718710	chr9p24.3-p24.1		3965456	Deletion									Array CGH		Not determined	GRCh37/hg19
EG7	A	SNV	46,XY	2	200245088	200245091	chr2:200245088-200245091	chr2q33.1	SATB2		Frameshift	ENST00000457245	NM_015265	594_595	ACC	A	199	Q>X	Heterozygous	Whole Exome Sequence		De Novo	GRCh37/hg19
EG7	B	CNV	46,XY	5	329960	489020	chr5:329960-489020	chr5p15.33		159061	Duplication									Array CGH		Paternal	GRCh36/hg18
EG8	A	CNV	46,XY	15	22765628	23082821	chr15:22765628-23082821	chr15q11.2		317194	Deletion									Array CGH		Not determined	GRCh37/hg19
EG9	A	SNV		16	89357096	89357096	chr16:89357096-89357096	chr16q24.3	ANKRD11		Frameshift	ENST00000301030	NM_013275	538	GG	G	180	D>TfsX48	Heterozygous	Whole Exome Sequence		De Novo	GRCh37/hg19
EG10	A	CNV	46,XX	22	22336298	22588389	chr22:22336298-22588389	chr22q11.22		252092	Deletion									Array CGH		Maternal	GRCh37/hg19
EG11	A	SNV	46,XX	X	41205569	41205569	chrX:41205569-41205569	chrXp11.4	DDX3X		Missense	ENST00000644876	NM_001356	1403	G	A	468	C>Y	Heterozygous	Whole Exome Sequence		De Novo	GRCh37/hg19
EG12	A	CNV	46,XX	22				chr22q11.2			Deletion									FISH	Vysis HIRA(-)	Not determined	

Appendix 2: Supplemental methods

Psychometric properties of DAWBA

The Development and Well-Being Assessment (DAWBA*)* is a validated research instrument [1,2] which has been used both in UK national and international surveys of child psychopathology [3,4,5]. It is available in 26 languages and is available digitally for 20 languages on https://www.dawba.info.
The DAWBA is organised into modules and was used to collect information on the child's family environment, caregiver wellbeing, schooling, emotional and behavioural adjustment, and specific mental health diagnoses. The surveys were rated according to DSM-5 criteria by psychiatrists using the same criteria as the recent national survey on children's mental health. Assessors were blind to the child's genetic condition. A sample of 147 DAWBAs were independently rated by IMAGINE-ID and DAWBA national study assessors. Substantial rates of inter-rater reliability were established ($\kappa > .67$, $p < .001$; See Table2).

Table2: Development and Well-Being Assessment *(*DAWBA) IMAGINE-ID and national Study inter-rater reliability (n=147)

**Diagnosis**	**Cohens Kappa**	***p***	**Level of Agreement**
**Any Disorder**	0·670	0·00	Substantial
**Any Emotional Disorder**	0·692	0·00	Substantial
**Any Anxiety Disorder**	0·785	0·00	Substantial
**ODD**	0·685	0·00	Substantial
**ADHD**	0·700	0·00	Substantial
**ASD**	0·798	0·00	Substantial

References:

[1]Goodman, R., Ford, T., Richards, H., Gatward, R., & Meltzer, H. (2000). The Development and Well-Being Assessment: description and initial validation of an integrated assessment of child and adolescent psychopathology. *Journal of child psychology and psychiatry, and allied disciplines*, *41*(5), 645–655.

[2]Goodman, A., Heiervang, E., Collishaw, S., & Goodman, R. (2011). The 'DAWBA bands' as an ordered-categorical measure of child mental health: description and validation in British and Norwegian samples. *Social psychiatry and psychiatric epidemiology*, *46*(6), 521–532.

[3] Ford, T., Goodman, R., & Meltzer, H. (2003). The British Child and Adolescent Mental Health Survey 1999: the prevalence of DSM-IV disorders. *Journal of the American Academy of Child and Adolescent Psychiatry*, *42*(10), 1203–1211. https://doi.org/10.1097/00004583-200310000-00011

[4] Heiervang, E., Goodman, A., & Goodman, R. (2008). The Nordic advantage in child mental health: separating health differences from reporting style in a cross-cultural comparison of psychopathology. *Journal of child psychology and psychiatry, and allied disciplines*, *49*(6), 678–685. https://doi.org/10.1111/j.1469-7610.2008.01882.x

[5] Emerson, E., & Hatton, C. (2007). Mental health of children and adolescents with intellectual disabilities in Britain. *The British journal of psychiatry : the journal of mental science*, *191*, 493–499. https://doi.org/10.1192/bjp.bp.107.038729

IMAGINE ID Supplemental Medical Questionnaire

The IMAGINE ID medical questionnaire is a bespoke questionnaire created specifically for the study but with reference to existing medical history questionnaires used in other research studies and in clinical practice. Certain questions are mapped to HPO/SNOMED terms for ease of analysis.

The medical questionnaire includes sections on:

- Ethnicity
- Family structure and history of similar problems
- Parental education and work
- Pregnancy details
- Birth details
- Neonatal development
- Infantile development
- Childhood development
- Current medical problems (by body system)
- Medication
- Current height, weight & head circumference
- Inheritance of genetic variant

See our online data dictionary for more information: https://imagine-id.org/wp-content/uploads/2019/04/Online-Data-dictionary-16.04.19-v2.pdf

ID: ____________

Welcome to the IMAGINE ID Medical Questionnaire.

We would like to ask you some more questions about your child's medical history.

The questionnaire looks longer than it is, it should only take **20 minutes** to complete. There are lots of skip rules so you will be able to jump through some of the sections fairly quickly.

Please provide an answer to all questions

If you have any questions or would prefer to complete this over the phone with us, please contact us on

Section 1. About your child

1. Child's first name ______________________________

2. What is your relationship to the child?

- ☐ Birth mother
- ☐ Birth father
- ☐ Adoptive mother
- ☐ Adoptive father
- ☐ Other (please specify) ____________________________________

3. Ethnic background of child

- ☐ White British
- ☐ White Irish
- ☐ Any other White background
- ☐ Mixed White and Black Caribbean
- ☐ Mixed White and Black African
- ☐ Mixed White and Asian
- ☐ Any other mixed background
- ☐ Indian
- ☐ Pakistani
- ☐ Bangladeshi
- ☐ Any other Asian background
- ☐ Black or Black British Caribbean
- ☐ Black or Black British African
- ☐ Any other Black background
- ☐ Chinese
- ☐ Any other ethnic group
- ☐ Unknown

4. Ethnic background of birth mother

- ☐ White British
- ☐ White Irish
- ☐ Any other White background
- ☐ Mixed White and Black Caribbean
- ☐ Mixed White and Black African
- ☐ Mixed White and Asian
- ☐ Any other mixed background
- ☐ Indian
- ☐ Pakistani
- ☐ Bangladeshi
- ☐ Any other Asian background
- ☐ Black or Black British Caribbean
- ☐ Black or Black British African
- ☐ Any other Black background
- ☐ Chinese
- ☐ Any other ethnic group
- ☐ Unknown

4. Ethnic background of birth father

- ☐ White British
- ☐ White Irish
- ☐ Any other White background
- ☐ Mixed White and Black Caribbean
- ☐ Mixed White and Black African
- ☐ Mixed White and Asian
- ☐ Any other mixed background
- ☐ Indian
- ☐ Pakistani
- ☐ Bangladeshi
- ☐ Any other Asian background
- ☐ Black or Black British Caribbean
- ☐ Black or Black British African
- ☐ Any other Black background
- ☐ Chinese
- ☐ Any other ethnic group
- ☐ Unknown

Section 2. About your family and household

1. How many brothers and sisters does your child have? ______

2. Please give the number of full and half brothers and sisters:

Number of full-brothers: _______

Number of half-brothers: _______

Number of full-sisters: _______

Number of half-sisters: _______

3. Does anyone else in the family have similar difficulties to your child?

- ☐ Yes
- ☐ No
- ☐ Don’t know/unsure

If yes, please specify who and what difficulties they have:

4. How many people are in the household (including yourself and child)? ___________

5. How many children in the household are under 16 (including your child)? __________

Section 3. About the parents' education and work

1. Do you know how old the birth mother was when your child was born?

- ☐ Yes, please specify ___________________
- ☐ No

2. Do you know how old the birth father was when your child was born?

- ☐ Yes, please specify ___________________
- ☐ No

3. What is the highest educational level of the birth mother?

- ☐ Did not complete compulsory education
- ☐ GCSEs or O-levels
- ☐ A-levels or Highers
- ☐ University degree
- ☐ Higher or postgraduate degree
- ☐ Vocational training, please specify ____
- ☐ Not known

4. What is the highest educational level of the birth father?

- ☐ Did not complete compulsory education
- ☐ GCSEs or O-levels
- ☐ A-levels or Highers
- ☐ University degree
- ☐ Higher or postgraduate degree
- ☐ Vocational training, please specify ____
- ☐ Not known

5. If applicable, what is the highest educational level of the adoptive parents?

6. What type of work does the birth mother do?

- ☐ Full time paid employment
- ☐ Part time paid employment
- ☐ Currently unemployed
- ☐ Full time training or education
- ☐ Part time training or education
- ☐ Full-time carer
- ☐ Voluntary work
- ☐ Not known
- ☐ Other, please specify ____

7. What type of work does the birth father do?

- ☐ Full time paid employment
- ☐ Part time paid employment
- ☐ Currently unemployed
- ☐ Full time training or education
- ☐ Part time training or education
- ☐ Full-time carer
- ☐ Voluntary work
- ☐ Not known
- ☐ Other, please specify ____

8. If applicable, what type of work do the adoptive parents do?

Section 4. About the pregnancy

1. Did the birth mother take any of the following in pregnancy? (Please select all that apply)

- ☐ Prescribed medication
- ☐ Anticonvulsant or antidepressant medication
- ☐ Other drugs
- ☐ Cigarettes (tobacco)
- ☐ Alcohol
- ☐ Not known
- ☐ **None of these**

If yes to any medication or other drugs taken during pregnancy, please give more details:

2. Did any of the following occur during pregnancy? (Please select all that apply)

- ☐ Maternal diabetes
- ☐ Maternal physical illness which needed medical attention
- ☐ Maternal mental illness which needed medical attention
- ☐ Assisted reproduction
- ☐ Multiple pregnancy
- ☐ Antenatal bleeding
- ☐ Abnormal nuchal translucency
- ☐ Abnormal ultrasound
- ☐ Abnormal amniocentesis
- ☐ Abnormal chorionic villous sampling
- ☐ Other (please specify)
- ☐ **None of these**

If yes to any of the above, please give more details: (For multiple pregnancy please specify type and how many are alive. For abnormal nuchal translucency please give measurements if known)

Section 5. About the birth

1. How many weeks pregnant was the mother when your child was born? _______

2. Was the birth caesarean (C-section)?

- ☐ Yes
- ☐ No
- ☐ Don't know/unsure

If yes, was this elective or emergency?

- ☐ Elective
- ☐ Emergency

3. Were there any problems with delivery?

- ☐ Yes
- ☐ No
- ☐ Don't know/unsure

If yes, what type of delivery problems?

- ☐ Forceps delivery
- ☐ Ventouse
- ☐ Foetal distress
- ☐ Cord around neck
- ☐ Other

4. Do you know your child's length at birth?

☐ Yes (please specify):

___________ Centimetres (cm) OR ___________ Inches (in)

☐ No

5. Do you know your child's weight at birth?

☐ Yes (please specify):

___________ Kilograms (kg) OR ___________ Pounds and ounces (lb and oz)

☐ No

6. Do you know your child's head circumference at birth?

☐ Yes (please specify):

___________ Centimetres (cm) OR ___________ Inches (in)

☐ No

Section 6: About neonatal development (from birth to 4 weeks old)

1. Was your child admitted to SCBU (Special Care Baby Unit) or NICU (Neonatal Intensive Care Unit)?

- ☐ Yes
- ☐ No
- ☐ Don't know/unsure

If yes, do you know how long they were there for?

- ☐ Yes (please specify)

_______ days OR _______ weeks OR _______ months

- ☐ No

2. Did your child have seizures as a newborn?

- ☐ Yes
- ☐ No
- ☐ Don't know/unsure

3. Was your child fed through a nasogastric (NG) tube as a newborn?

- ☐ Yes
- ☐ No
- ☐ Don't know/unsure

4. Was your child ventilated as a newborn?

- ☐ Yes
- ☐ No
- ☐ Don't know/unsure

If yes, do you know how long they were they ventilated for?

________ ☐ Days ☐ Weeks ☐ Months OR ☐ Unsure

Section 7. About your child's infancy

	Yes	No	Don't know/ unsure
1. Did your child have decreased tone (were they floppy) as a baby?	☐	☐	☐
2. Did your child have increased tone (were they stiff), spasticity or cerebral palsy as a baby?	☐	☐	☐
3. Did your child have severe colic as a baby?	☐	☐	☐
4. Was your child irritable, miserable or difficult to settle as a baby?	☐	☐	☐
5. Was your child lethargic or overly sleepy as a baby?	☐	☐	☐

Section 8. About your child's development

1. Did your child have failure to thrive in infancy?

☐ Yes
☐ No
☐ Don't know/unsure

2. Did your child have feeding problems in childhood?

☐ Yes
☐ No
☐ Don't know/unsure

3. Was your child fed with a tube in childhood?

☐ Yes
☐ No
☐ Don't know/unsure

If yes, how long were they fed with a tube for?

__________ ☐ Days ☐ Weeks ☐ Months ☐ Don't know/unsure

4. Did your child have a social smile by 2 months old?

☐ Yes
☐ No
☐ Don't know/unsure

If no, at what age did your child have a social smile? (If you're unsure please give your best guess)

__________ ☐ Weeks ☐ Months ☐ Years

5. Can your child sit independently?

☐ Yes
☐ No
☐ Don't know/unsure

If yes, at what age could they do this? (If you're unsure please give your best guess)

__________ ☐ Weeks ☐ Months ☐ Years

6. Can or could your child crawl?

☐ Yes
☐ No
☐ Don't know/unsure

If yes, how can or could they crawl? (please select all that apply)

☐ Rolling
☐ Bottom shuffling
☐ Commando-style
☐ Hands and knees
☐ Not known

At what age could they do this? (If you're unsure please give your best guess)

__________ ☐ Weeks ☐ Months ☐ Years

	Yes	No	**If yes,** please give the age your child could do this, if you're unsure please give your best guess
7. Can your child walk independently?	☐	☐	_____ ☐ Weeks ☐ Months ☐ Years
8. Can your child run?	☐	☐	_____ ☐ Weeks ☐ Months ☐ Years
9. Can your child hop?	☐	☐	_____ ☐ Weeks ☐ Months ☐ Years
10. Can your child ride a bike without stabilizers?	☐	☐	_____ ☐ Weeks ☐ Months ☐ Years
11. Can your child do up buttons?	☐	☐	_____ ☐ Weeks ☐ Months ☐ Years
12. Can your child get dressed by themselves?	☐	☐	_____ ☐ Weeks ☐ Months ☐ Years
13. Does your child have a sense of danger?	☐	☐	
14. Can your child speak?	☐	☐	_____ ☐ Weeks ☐ Months ☐ Years

15. What was your child's language level at 4 years old? (Please select one)

- ☐ No speech and language
- ☐ Minimal (single words)
- ☐ Simple sentences
- ☐ Slightly delayed
- ☐ Enough to communicate their needs
- ☐ Average for their age
- ☐ Not known

16. Has your child lost any developmental skills that they used to have?

- ☐ Yes
- ☐ No
- ☐ Don't know/unsure

If yes, please give detail about any loss of developmental skills:

17. Does your child have a Statement of Special Educational Need or Education and Health Care Plan?

- ☐ Yes
- ☐ No

18. Does your child receive a Disability Living Allowance?

- ☐ Yes
- ☐ No

If yes, please answer the following questions:

18a. What level is the Care component of the Allowance?

☐ Not eligible ☐ Lowest ☐ Middle ☐ Highest
☐ Don't know/unsure

18b. What level is the Mobility component of the Allowance?

☐ Not eligible ☐ Lowest ☐ Middle ☐ Highest
☐ Don't know/unsure

Section 9. About medical problems your child may have

This section asks about any medical problems your child currently has or has previously had.
- **If you answer 'Yes', please continue and answer further questions so that you can give more detail about this.**
- **If you answer 'No' or 'Don't know/unsure' you can move onto the next question.**

1. Has your child ever had any hearing problems?

☐ Yes
☐ No
☐ Don't know/unsure

If yes, please tick all that apply:

		Do they still have this hearing problem?	
		**Yes**	**No**
☐	Hearing Aid	☐	☐
☐	Glue ear	☐	☐
☐	Grommets	☐	☐
☐	Any other hearing problems	☐	☐

Please give detail about any hearing problems:

2. Has your child ever had any eye problems?

☐ Yes
☐ No
☐ Don't know/unsure

If yes, please tick all that apply:

		Do they still have this eye problem?	
		**Yes**	**No**
☐	Glasses	☐	☐
☐	Squint	☐	☐
☐	Cataracts	☐	☐
☐	Any other eye problems	☐	☐

Please give detail about any eye problems:

3. Has your child ever had a cleft lip or palate?

- ☐ Yes
- ☐ No
- ☐ Don't know/unsure

If yes, please tick all that apply:

		**Do they still have this problem?**	
		**Yes**	**No**
☐	Cleft lip	☐	☐
☐	Cleft palate	☐	☐
☐	Difficulty swallowing	☐	☐
☐	Speech and language difficulty	☐	☐
☐	Any other cleft or speech and language problems	☐	☐

Please give detail about any other cleft or speech and language problems:

4. Has your child ever had any muscle or movement problems?

- ☐ Yes
- ☐ No
- ☐ Don't know/unsure

If yes, please tick all that apply:

		**Do they still have this muscle/movement problem?**	
		**Yes**	**No**
☐	Clumsy	☐	☐
☐	Low tone/floppy	☐	☐
☐	High tone/stiff/spasticity	☐	☐
☐	Tremor	☐	☐
☐	Ataxia	☐	☐
☐	Dystonia	☐	☐
☐	Fine motor skills problems	☐	☐
☐	Cerebral palsy	☐	☐
☐	Any other muscle or movement problems	☐	☐

Please give detail about any other muscle or moment problems:

5. Has your child ever had any brain damage caused by an infection or trauma?

- ☐ Yes
- ☐ No
- ☐ Don't know/unsure

If yes, please tick all that apply:

- ☐ Meningitis
- ☐ Encephalitis
- ☐ Head injury
- ☐ Any other brain disease

Please give details:

6. Has your child had any brain/MRI/CT/EEG scans?

- ☐ Yes
- ☐ No
- ☐ Don't know/unsure

If yes, please tick all that apply:

		Was the scan:		
		**Normal**	**Abnormal**	**Unsure**
☐	MRI scan	☐	☐	☐
☐	CT scan	☐	☐	☐
☐	EEG	☐	☐	☐
☐	Brain scan	☐	☐	☐
☐	Any other scan	☐	☐	☐

If yes to any scans, please give detail about any other muscle or moment problems:

7. Does your child have a brain malformation or neurodegenerative disease?

- ☐ Yes
- ☐ No
- ☐ Don't know/unsure

If yes, please describe if known:

8. Has your child ever had any seizures?

☐ Yes
☐ No
☐ Don't know/unsure

<u>If yes</u>, please answer the following:

8a. Are the seizures currently under control?

☐ Yes
☐ No
☐ Don't know/unsure

8b. Please tick all types of seizures that your child has had:

		Do they still have these seizures?	
		**Yes**	**No**
☐	Febrile seizures	☐	☐
☐	Grand mal/generalised tonic clonic seizures	☐	☐
☐	Petit mal/absence seizures	☐	☐
☐	Infantile spasms	☐	☐
☐	Atonic/drop attacks	☐	☐
☐	Complex partial seizures	☐	☐
☐	Simple partial/focal seizures	☐	☐
☐	Convulsive/non-convulsive status epilepticus	☐	☐
☐	Electrical status epilepticus in sleep	☐	☐
☐	Unsure of type	☐	☐
☐	Any other seizures	☐	☐

Please give details of any other seizures:

9. Has your child ever had any stomach and/or gut problems?

☐ Yes
☐ No
☐ Don't know/unsure

If yes, please tick all that apply:

		Do they still have these problems? Yes	No
☐	Reflux	☐	☐
☐	Severe constipation	☐	☐
☐	Gastroschisis	☐	☐
☐	Omphalocele	☐	☐
☐	Hirschsprung disease	☐	☐
☐	Malrotation of the gut	☐	☐
☐	Tracheoesophageal fistula	☐	☐
☐	Duodenal atresia	☐	☐
☐	Anal atresia	☐	☐
☐	Any other stomach and/or gut problems	☐	☐

Please give detail about any other stomach and/or gut problems:

10. Has your child ever had any breathing or chest problems?

☐ Yes
☐ No
☐ Don't know/unsure

If yes, please tick all that apply:

		Do they still have these breathing or chest problems? Yes	No
☐	Recurrent chest infections	☐	☐
☐	Asthma	☐	☐
☐	Bronchiectasis	☐	☐
☐	Any other breathing or chest problems	☐	☐

Please give detail about any other muscle or moment problems:

11. Has your child ever had any heart problems?

- ☐ Yes
- ☐ No
- ☐ Don't know/unsure

If yes, please tick all that apply:

- ☐ Congenital structural heart defect
- ☐ Heart rhythm disturbance
- ☐ Any other heart problem

Please give details:

12. Has your child ever had any kidney problems?

- ☐ Yes
- ☐ No
- ☐ Don't know/unsure

**If yes,** please tick all that apply:		**Do they still have these kidney problems?**	
		**Yes**	**No**
☐	Urinary reflux	☐	☐
☐	Recurrent urinary tract infections	☐	☐
☐	Renal cysts	☐	☐
☐	Renal tumors	☐	☐
☐	Abnormal kidney shape	☐	☐
☐	Abnormal kidney function	☐	☐
☐	Any other kidney problems	☐	☐

Please give detail about any other kidney problems:

13. Has your child ever had any genital problems?

☐ Yes
☐ No
☐ Don't know/unsure

If yes, please tick all that apply:

		Do they still have these genital problems?	
		**Yes**	**No**
☐	Ambiguous genitalia	☐	☐
☐	Undescended testes	☐	☐
☐	Hypospadias	☐	☐
☐	Any other genital problems	☐	☐

Please give detail about any other genital problems:

14. Has your child ever had any skeletal or bone problems?

☐ Yes
☐ No
☐ Don't know/unsure

If yes, please tick all that apply:

		Do they still have these skeletal or bone problems?	
		**Yes**	**No**
☐	Polydactyly	☐	☐
☐	Abnormal hands	☐	☐
☐	Abnormal feet	☐	☐
☐	Vertebral anomalies	☐	☐
☐	Scoliosis	☐	☐
☐	Chest wall problems	☐	☐
☐	Any other bone problems	☐	☐

Please give details about any other bone problems:

15. Has your child ever had any growth or hormone problems?

☐ Yes
☐ No
☐ Don't know/unsure

**If yes,** please tick all that apply:		**Do they still have these growth or hormone problems?** Yes	No
☐	Short stature	☐	☐
☐	Growth hormone deficiency	☐	☐
☐	Failure to thrive	☐	☐
☐	Early puberty	☐	☐
☐	Insulin-dependent diabetes	☐	☐
☐	Abnormal kidney function	☐	☐
☐	Any other hormone problems	☐	☐

Please give detail about any other hormone problems:

16. Has your child gone through puberty? (Please select one)

☐ Yes
☐ Currently ongoing
☐ No
☐ Don't know/unsure

Please give details:

17. Has your child ever had any sleep problems?

- ☐ Yes
- ☐ No
- ☐ Don't know/unsure

**If yes,** please tick all that apply:		**Do they still have these sleep problems?**	
		**Yes**	**No**
☐	Trouble getting to sleep	☐	☐
☐	Early waking	☐	☐
☐	Multiple interruptions to sleep	☐	☐
☐	Insomnia	☐	☐
☐	Hypersomnia	☐	☐
☐	Night terrors	☐	☐
☐	Sleepwalking	☐	☐
☐	Any other sleep problems	☐	☐

Please give detail about any other sleep problems:

18. How many hours does your child normally sleep each night? ________

19. If you would like to give any further details of your child's sleep problems, please do so:

20. Has your child ever had any food or eating problems?

- ☐ Yes
- ☐ No
- ☐ Don't know/unsure

If yes, please tick all that apply:

		Do they still have these food or eating problems?	
		**Yes**	**No**
☐	Binge eating	☐	☐
☐	Parents need to restrict access to food	☐	☐
☐	Eating non-food items	☐	☐
☐	Extreme food rituals, habits or preferences	☐	☐
☐	Any other food behaviour problems	☐	☐

Please give detail about any food or eating problems:

21. Does your child have regular medical/outpatient appointments?

- ☐ Yes
- ☐ No

If yes, what speciality, what is the name of their doctor and which hospital are they seen at?

22. If your child has any other serious medical or dental problems, please let us know:

Section 10. About any medication your child may take

This section asks about any medication your child is currently taken or has previously taken.

- **If you answer 'Yes', please continue to answer further questions so we can learn more about this medication.**
- **If you answer 'No' or 'Don't know/unsure' you can move onto the next question.**

1. Is your child currently taking or have they taken any medication for sleep problems?

☐ Yes
☐ No
☐ Don't know/unsure

<u>If yes,</u> please answer the following questions:

1a. Please choose the name of the medication:

☐ Melatonin/Bio-Melatonin/Circadin
☐ Promethazine Hydrochloride/ Phenergan
☐ Zopiclone/Zimovane
☐ Other, please state ____

1b. Are they currently on this medication?

☐ Yes
☐ No

1c. How long have they been taking this medication?

________ ☐ Weeks ☐ Months ☐ Years

1d. Did this medication work?

☐ No
☐ A little
☐ A lot
☐ Don't know/unsure

2. Is your child currently taking or have they taken any medication for anxiety or low mood problems?

☐ Yes
☐ No
☐ Don't know/unsure

<u>If yes,</u> please answer the following questions:

2a. Please choose the name of the medication:

☐ Citalopram Hydrobromide/Cipramil/Citalopram Hydrochloride
☐ Clomipramine Hydrochloride/Anafranil
☐ Fluoxetine Hydrochloride/Olena/Oxactin/Prozac/Prozit
☐ Mirtazapine/Zispin
☐ Sertraline Hydrochloride/Lustral
☐ Other, please state ____

2b. Are they currently on this medication?

☐ Yes
☐ No

2c. How long have they been taking this medication?

________ ☐ Weeks ☐ Months ☐ Years

2d. Did this medication work?

☐ No
☐ A little
☐ A lot
☐ Don't know/unsure

3. Is your child currently taking or have they taken any medication for behavioural problems (irritation, aggression, agitation)?

☐ Yes
☐ No
☐ Don't know/unsure

<u>If yes,</u> please answer the following questions:

3a. Please choose the name of the medication:

☐ Risperidone/ Risperdal
☐ Aripiprazole/Abilify
☐ Olanzapine/Zalasta/Zyprexa/Zypadhera
☐ Diazepam/Lorazepam
☐ Fluoxetine Hydrochloride/Sertraline Hydrochloride
☐ Other, please state ____

3b. Are they currently on this medication?

☐ Yes
☐ No

3c. How long have they been taking this medication?

________ ☐ Weeks ☐ Months ☐ Years

3d. Did this medication work?

☐ No
☐ A little
☐ A lot
☐ Don't know/unsure

4. Is your child currently taking or have they taken any medication for hyperactivity/ADHD?

☐ Yes
☐ No
☐ Don't know/unsure

<u>If yes,</u> please answer the following questions:

4a. Please choose the name of the medication:

☐ Atomoxetine Hydrochloride/Strattera
☐ Dexamfetamine Sulfate/Adderall/Dexamfet/Dexedrine
☐ Methylphenidate HCl/Concerta/Equasym/Matoride/Medikinet/Ritalin/Tranquilyn
☐ Lisdexamfetamine Dimesylate/Elvanse/Vyvanse
☐ Other, please state ____

4b. Are they currently on this medication?

☐ Yes
☐ No

4c. How long have they been taking this medication?

________ ☐ Weeks ☐ Months ☐ Years

4d. Did this medication work?

☐ No
☐ A little
☐ A lot
☐ Don't know/unsure

5. Is your child currently taking or have they taken any medication for tics?

☐ Yes
☐ No
☐ Don't know/unsure

If yes, please answer the following questions:

5a. Please choose the name of the medication:

☐ Risperidone/Risperdal
☐ Aripiprazole/Abilify
☐ Clonidine Hydrochloride
☐ Guanfacine/Intuniv
☐ Other, please state ______________________________

5b. Are they currently on this medication?

☐ Yes
☐ No

5c. How long have they been taking this medication?

________ ☐ Weeks ☐ Months ☐ Years

5d. Did this medication work?

☐ No
☐ A little
☐ A lot
☐ Don't know/unsure

6. Is your child currently taking or have they taken any medication for bedwetting problems?

☐ Yes
☐ No
☐ Don't know/unsure

If yes, please answer the following questions:

6a. Please choose the name of the medication:

☐ Desmopressin Acetate/DDAVP
☐ Imipramine Hydrochloride
☐ Oxybutynin/Cystrin
☐ Tolterodine/Efflosomyl
☐ Other, please state ______________________________

6b. Are they currently on this medication?

☐ Yes
☐ No

6c. How long have they been taking this medication?

________ ☐ Weeks ☐ Months ☐ Years

6d. Did this medication work?

☐ No
☐ A little
☐ A lot
☐ Don't know/unsure

7. Is your child currently taking or have they taken any medication for seizures?

- ☐ Yes
- ☐ No
- ☐ Don't know/unsure

<u>If yes,</u> please answer the following questions:

7a. Please choose the name of the medication:

- ☐ Carbamazepine/Carbagen/Epimaz/Tegretol
- ☐ Clobazam/Frisium/Tapclob
- ☐ Clonazepam/Rivotril
- ☐ Eslicarbazepine Acetate/Zebinix
- ☐ Ethosuximide/Emeside/Zarontin
- ☐ Felbamate/Felbatol/Taloxa
- ☐ Gabapentin/Neurontin
- ☐ Lacosamide/Vimpat
- ☐ Lamotrigine/Lamictal
- ☐ Levetiracetam/Desitrend/Keppra/Matever
- ☐ Levetiracetam/Desitrend/Keppra/Matever
- ☐ Midazolam Hydrochloride/ Buccolam/Midazolam Maleate/Dormicum
- ☐ Oxcarbazepine/Trileptal
- ☐ Paraldehyde
- ☐ Perampanel/Fycompa
- ☐ Phenobarbital
- ☐ Phenytoin Sodium/Epanutin
- ☐ Pregabalin/Lyrica
- ☐ Primidone/Mysoline
- ☐ Retigabine/Trobalt
- ☐ Rufinamide/Inovelon
- ☐ Sodium Valporate/Epilim/ Episenta/Epival/Orlept
- ☐ Stiripentol/Diacomit
- ☐ Sultiame/Ospolot
- ☐ Tiagabine/Gabitril
- ☐ Topiramate/Topamax
- ☐ Valproic Acid/Convulex
- ☐ Vigabatrin/Sabril
- ☐ Zonisamide/Zonegran
- ☐ Other, please state ________________________

7b. Are they currently on this medication?

- ☐ Yes
- ☐ No

7c. How long have they been taking this medication?

________ ☐ Weeks ☐ Months ☐ Years

7d. Did this medication work?

- ☐ No
- ☐ A little
- ☐ A lot
- ☐ Don't know/unsure

8. Is your child currently taking or have they taken any other medications?

- ☐ Yes
- ☐ No
- ☐ Don't know/unsure

<u>If yes,</u> please answer the following questions:

8a. Please give details about this medication:

8b. Are they currently on this medication?

- ☐ Yes
- ☐ No

8c. How long have they been taking this medication?

________ ☐ Weeks ☐ Months ☐ Years

8d. Did this medication work?

- ☐ No
- ☐ A little
- ☐ A lot
- ☐ Don't know/unsure

Section 11. And finally... about your child now

The next section is about your child now. You can either measure your child now or you can use measurements taken recently - just let us know when the measurements were taken.

1. Do you know your child's height now?

☐ Yes
☐ No

If yes, please answer the following questions:

1a. What is your child's height now?

_________ Feet and inches (ft and in)

OR _________ Centimetres (cm)

1b. What date was this measured? Or your best guess.

____

2. Do you know your child's weight now?

☐ Yes
☐ No

If yes, please answer the following questions:

2a. What is your child's weight now?

_________ Kilograms (kg)

OR _________ Stone and pounds (st and lb)

OR _________ Pounds (lb)

2b. What date was this measured? Or your best guess.

____

3. Do you know your child's head circumference now?

☐ Yes
☐ No

If yes, please answer the following questions:

3a. What is your child's head circumference now?

_________ Inches (in)

OR _________ Centimetres (cm)

3b. What date was this measured? Or your best guess.

____

4. It can sometimes be helpful to know how a genetic change happened. Thinking about your child's genetic condition:

☐ It is new in the family (de novo)
☐ It is inherited from a parent (familial)
☐ Parents were not tested
☐ Don't know/unsure
☐ Prefer not to say

That’s the end of the Medical Questionnaire! Thank you very much for completing this for us, your contribution is hugely valuable and will help other families who are affected by rare genetic conditions.

Appendix 4: Supplementary Tables and Figures

Table 3: N numbers for analyses

N numbers	SDQ	DAWBA	ABAS	Medical	IMD by household	Documented age of diagnosis
**Table 1**	2397	Diagnoses 2186	1238	1277	2142	2349
**Table 3**	1106	Diagnoses 1021	549	..	961	1098

Numbers of completed questionnaires from participants included in analyses

Table 4: Cross-tabulation of variant type and variant inheritance

Inheritance<br>Variant Type	familial	de novo	unknown
**CNV**	564	541	665
**SNV**	81	399	147

Table 5: IMD distributions by household

IMD Quintile<br>No. (%)	National sample	Recruited cohort<br>N=2476	Assessment completers<br>N= 2142
1 – most deprived	20%	536 (21·6)	431 (20·1)
2	20%	458 (18·5)	406 (19)
3	20%	471 (19)	407 (19)
4	20%	477 (19·3)	427 (19·9)
5 – least deprived	20%	534 (21·6)	471 (22)

Of 2770 participants recruited to IMAGINE, IMD scores were available on 2642 individuals from 2476 households. Of the 2397 children for whom measures of mental health are available, postcodes linked to IMD scores were available on 2277 UK participants from 2142 households.

Table 6: SDQ subscale data (n=2397)

SDQ severity band<br>No. (%)	Emotional problems	Conduct problems	Hyperactivity/ Inattention problems	Peer problems	Pro-social ability	Impact
**Close to average**	929 (38·8)	990 (41·3)	424 (17·7)	499 (20·8)	600 (25)	206 (8·6)
**Slightly raised**	284 (11·8)	420 (17·5)	496 (20·7)	322 (13·4)	273 (11·4)	112(4·7)
**High**	509 (21·2)	556 (23·2)	401 (16·7)	367 (15·3)	274 (11·4)	145 (6)
**Very high**	675 (28·2)	431 (18)	1076 (44·9)	1209 (50·4)	1250 (52·1)	1934 (80·7)

Table 7: Difficulties with seizures, epilepsy, muscle and movement problems reported in medical questionnaire (n=1277)

Area of difficulty	N (%)
**Types of seizure (n=355)**	
Petit mal / Absence seizures	148 (41·7)
Grand mal / Generalised tonic	120 (33·8)
Febrile seizures	94 (26·5)
Simple partial / focal seizures	57 (16·1)
Any other seizure type	51 (14·4)
Partial complex seizures	46 (13)
Atonic / drop attacks	38 (10·7)
Convulsive / non-convulsive status epilepticus	37 (10·4)
Infantile spasms	22 (6·2)
Electrical status epilepticus in sleep	12 (3·4)
Unsure of seizure type	69 (19·4)
**Number of seizure types (n=355)**	
1	147 (41·4)
2	76 (21·4)
3	33 (9·3)
4+	44 (12·4)
unsure	55 (15·5)
**Types of muscle problems (n=814)**	
High tone	98 (7)
Low tone	492 (3)
Dystonia	33 (2·6)
**Types of movement & co-ordination problems (n=814)**	
Fine motor skill difficulties	588 (46)
Clumsy	432 (33·8)
Tremor	41 (93)
Ataxia	57 (4·5)
Cerebral palsy	24 (1.9)
**Other muscle or movement problems (n=814)**	
Other	316 (24·7)

Figure 1 : Scatter plot of IMD score and SDQ scores by inheritance in CNV group
IMD scores were plotted against SDQ scores and grouped by inheritance of the genetic anomaly. Black line (*de novo* inheritance) and dashed line (familial inheritance) do not intersect, indicating that deprivation and variant inheritance independently contributed to behaviour difficulties.

•= de novo variant
o = familial variant
Black line = *de novo* inheritance
Dashed line = familial inheritance

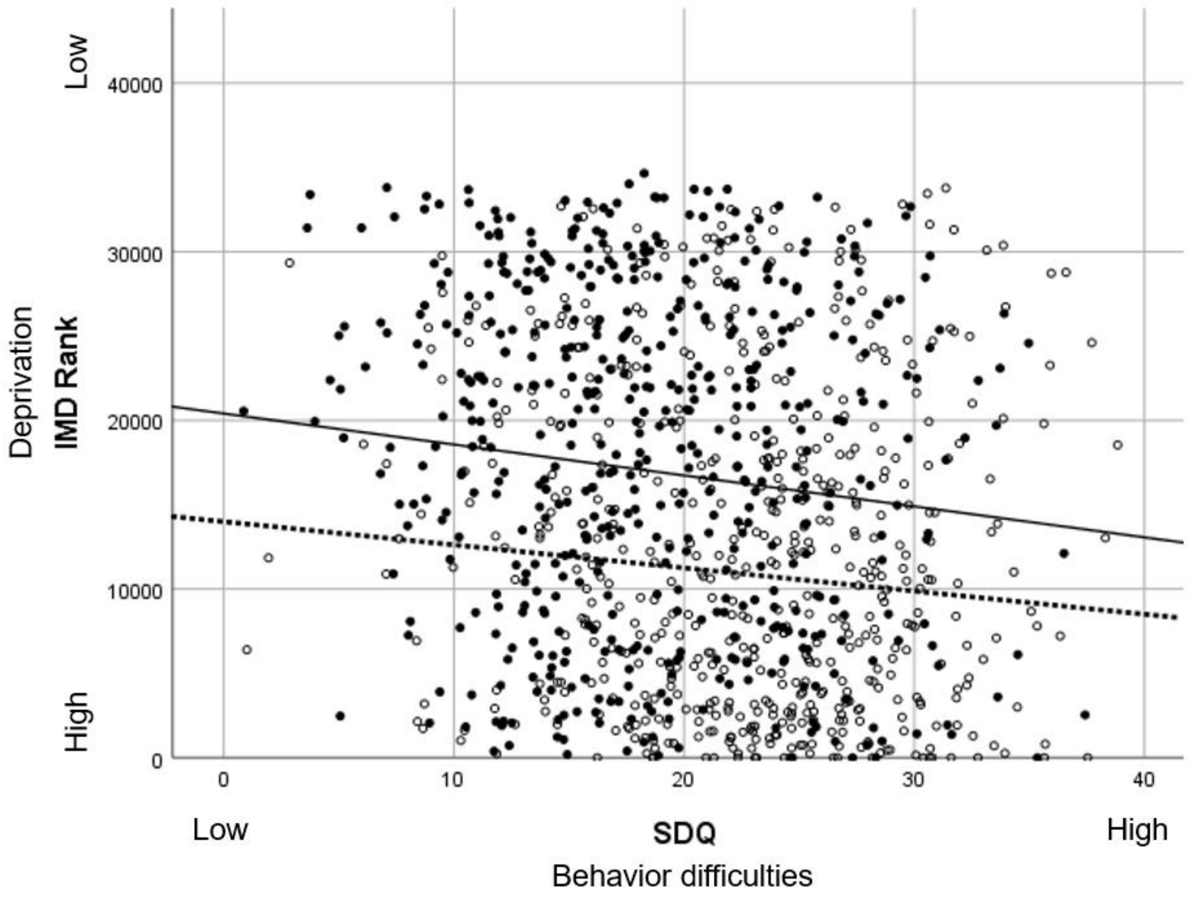

Appendix 5: IMAGINE Study members

Surname	Initials	First Name	Title	Institution
**Raymond**	F L	F Lucy	Professor	Department of Medical Genetics, University of Cambridge, UK
**Dewhurst**	E	Eleanor	Mrs	Department of Medical Genetics, University of Cambridge, UK
**Lafont**	A	Amy	Ms	Department of Medical Genetics, University of Cambridge, UK
**Timur**	H	Husniye	Ms	Department of Medical Genetics, University of Cambridge, UK
**Wicks**	F	Francesca	Mrs	Department of Medical Genetics, University of Cambridge, UK
**Ye**	Z	Zheng	Dr	Department of Medical Genetics, University of Cambridge, UK
**Baker**	K	Kate	Dr	Department of Medical Genetics, University of Cambridge, UK
**Walker**	N	Neil	Dr	Department of Medical Genetics, University of Cambridge, UK
**Wallwork**	S	Sarah	Ms	Department of Medical Genetics, University of Cambridge, UK
**Skuse**	D	David	Professor	Great Ormond Street Institute of Child Health, University College London, UK
**Denaxas**	S	Spiros	Dr	Institute of Health Informatics, University College London, London, UK
**Mandy**	W	William	Dr	Division of Psychology & Language Sciences, University College London, UK
**Wolstencroft**	J	Jeanne	Dr	Great Ormond Street Institute of Child Health, University College London, UK
**Davies**	S	Sarah	Ms	Great Ormond Street Institute of Child Health, University College London, UK
**Erwood**	M	Marie	Ms	Great Ormond Street Institute of Child Health, University College London, UK
**Juj**	M	Manoj	Mr	Great Ormond Street Institute of Child Health, University College London, UK
**Kerry**	E	Eleanor	Ms	Great Ormond Street Institute of Child Health, University College London, UK
**Lucock**	A	Anna	Ms	Great Ormond Street Institute of Child Health, University College London, UK
**Printzlau**	F	Frida	Ms	Great Ormond Street Institute of Child Health, University College London, UK

**Srinivasan**	R	Ramya	Dr	Great Ormond Street Institute of Child Health, University College London, UK
**Walker**	S	Susan	Dr	Great Ormond Street Institute of Child Health, University College London, UK
**Watkins**	A	Alice	Ms	Great Ormond Street Institute of Child Health, University College London, UK
**Coscini**	N	Nadia	Dr	Great Ormond Street Institute of Child Health, University College London, UK
**Fatih**	N	Nasrtullah	Mr	Great Ormond Street Institute of Child Health, University College London, UK
**Nayana**	L	Lahiri	Ms	Great Ormond Street Institute of Child Health, University College London, UK
**Denyer**	H	Hayley	Ms	Great Ormond Street Institute of Child Health, University College London, UK
**Andrews**	S	Sophie	Ms	MRC Centre for Neuropsychiatric Genetics and Genomics, Division of Psychological Medicine and Clinical Neurosciences, Cardiff University, UK
**Chawner**	SJRA	Samuel	Dr	MRC Centre for Neuropsychiatric Genetics and Genomics, Division of Psychological Medicine and Clinical Neurosciences, Cardiff University, UK
**Cuthbert**	A	Andrew	Dr	MRC Centre for Neuropsychiatric Genetics and Genomics, Division of Psychological Medicine and Clinical Neurosciences, Cardiff University, UK
**Challenger**	A	Aimee	Ms	MRC Centre for Neuropsychiatric Genetics and Genomics, Division of Psychological Medicine and Clinical Neurosciences, Cardiff University, UK
**Hall**	J	Jeremy	Professor	MRC Centre for Neuropsychiatric Genetics and Genomics, Division of Psychological Medicine and Clinical Neurosciences, Cardiff University, UK
**Lewis**	N	Nicola	Ms	MRC Centre for Neuropsychiatric Genetics and Genomics, Division of Psychological Medicine and Clinical Neurosciences, Cardiff University, UK
**Owen**	MJ	Michael	Professor Sir	MRC Centre for Neuropsychiatric Genetics and Genomics, Division of Psychological Medicine and Clinical Neurosciences, Cardiff University, UK
**Ray**	S	Sinead	Ms	MRC Centre for Neuropsychiatric Genetics and Genomics, Division of Psychological Medicine and Clinical Neurosciences, Cardiff University, UK
**Sopp**	M	Matthew	Mr	MRC Centre for Neuropsychiatric Genetics and Genomics, Division of Psychological Medicine and Clinical Neurosciences, Cardiff University, UK
**Moss**	H	Hayley	Ms	MRC Centre for Neuropsychiatric Genetics and Genomics, Division of Psychological Medicine and Clinical Neurosciences, Cardiff University, UK
**van den Bree**	MBM	Marianne	Professor	MRC Centre for Neuropsychiatric Genetics and Genomics, Division of Psychological Medicine and Clinical Neurosciences, Cardiff University, UK

**Holmans**	P	Peter	Professor	MRC Centre for Neuropsychiatric Genetics and Genomics, Division of Psychological Medicine and Clinical Neurosciences, Cardiff University, UK
**Bowen**	S	Samantha	Ms	MRC Centre for Neuropsychiatric Genetics and Genomics, Division of Psychological Medicine and Clinical Neurosciences, Cardiff University, UK
**Bradley**	K	Karen	Mrs	MRC Centre for Neuropsychiatric Genetics and Genomics, Division of Psychological Medicine and Clinical Neurosciences, Cardiff University, UK
**Birch**	B	Philippa	Ms	MRC Centre for Neuropsychiatric Genetics and Genomics, Division of Psychological Medicine and Clinical Neurosciences, Cardiff University, UK
**Tong**	M	Molly	Ms	MRC Centre for Neuropsychiatric Genetics and Genomics, Division of Psychological Medicine and Clinical Neurosciences, Cardiff University, UK
**Ford**	T	Tasmin	Professor	Department of Psychiatry, University of Cambridge
**Searle**	B	Beverly	Dr	Unique Charity, UK
**Wynn**	S	Sarah	Dr	Unique Charity, UK
**Robertson**	L	Lisa	Dr	Aberdeen Royal Infirmary Genetics Service
**Berg**	J	Jonathan	Dr	Ninewells Hospital Dundee Genetics Service
**Lampe**	A	Anne	Professor	Western General Hospital Edinburgh Genetics Service
**Joss**	S	Shelagh	Dr	Glasgow Genetics Centre, Glasgow
**Brennan**	P	Paul	Dr	Northern Genetics Service, Newcastle
**Kraus**	A	Alison	Dr	Yorkshire Regional Genetics Service - Clinical Genetics
**Weber**	A	Astrid	Dr	Cheshire & Merseyside Regional Genetic Service
**Rawson**	M	Myfanwy	Ms	Manchester Centre for Genomic Medicine
**Quarrell**	O	Oliver	Dr	Sheffield Genetic Services
**Vasudevan**	P	Pradeep	Dr	Leicestershire Genetics Centre, Leicester
**Harrison**	R	Rachel	Dr	Nottingham Regional Genetics Service
**Williams**	D	Denise	Dr	West Midlands Regional Genetics Service, Birmingham
**Maher**	E	Eamonn	Professor	East Anglian Medical Genetics Service, Cambridge
**Kini**	U	Usha	Dr	Oxford Genetics Service
**Clowes**	V	Virginia	Dr	London North West Thames Regional Genetics Service
**Van Dijk**	F	Fleur	Dr	London North West Thames Regional Genetics Service
**Gurasashvilli**	J	Jana	Dr	London North East Thames Regional Genetics Service - Great Ormond Street Hospital, London
**Mansour**	S	Sahar	Dr	London South West Thames Regional Genetics Service, St Georges Hospital, Tooting, London

**Holder-Espinasse**	M	Muriel	Dr	London South East Thames Regional Genetics Service Guy's Hospital, London
**Watford**	A	Amy	Dr	Bristol Clinical Genetics Service, Bristol
**Rankin**	J	Julia	Dr	Peninsula Genetics Service, Exeter
**Baralle**	D	Diana	Dr	Wessex Clinical Genetics Service
**Procter**	A	Annie	Dr	All Wales Regional Genetics Service

Appendix 6: STROBE reporting guidelines

	Item No	Recommendation	Page No
**Title and abstract**	1	(*a*) Indicate the study's design with a commonly used term in the title or the abstract	1
		(*b*) Provide in the abstract an informative and balanced summary of what was done and what was found	1
**Introduction**			
Background/rationale	2	Explain the scientific background and rationale for the investigation being reported	1-2
Objectives	3	State specific objectives, including any prespecified hypotheses	1-2
**Methods**			
Study design	4	Present key elements of study design early in the paper	2-4
Setting	5	Describe the setting, locations, and relevant dates, including periods of recruitment, exposure, follow-up, and data collection	2-4
Participants	6	(*a*) Give the eligibility criteria, and the sources and methods of selection of participants. Describe methods of follow-up	2
		(*b*) For matched studies, give matching criteria and number of exposed and unexposed	N/A
Variables	7	Clearly define all outcomes, exposures, predictors, potential confounders, and effect modifiers. Give diagnostic criteria, if applicable	3-4
Data sources/ measurement	8*	For each variable of interest, give sources of data and details of methods of assessment (measurement). Describe comparability of assessment methods if there is more than one group	3-4, Appendix
Bias	9	Describe any efforts to address potential sources of bias	9-9
Study size	10	Explain how the study size was arrived at	Figure 1
Quantitative variables	11	Explain how quantitative variables were handled in the analyses. If applicable, describe which groupings were chosen and why	N/A
Statistical methods	12	(*a*) Describe all statistical methods, including those used to control for confounding	4-5
		(*b*) Describe any methods used to examine subgroups and interactions	4-5
		(*c*) Explain how missing data were addressed	Figure 1, Appendix
		(*d*) If applicable, explain how loss to follow-up was addressed	N/A
		(*e*) Describe any sensitivity analyses	Appendix
**Results**			
Participants	13*	(a) Report numbers of individuals at each stage of study—eg numbers potentially eligible, examined for eligibility, confirmed eligible, included in the study, completing follow-up, and analysed	Figure 1
		(b) Give reasons for non-participation at each stage	

		(c) Consider use of a flow diagram	
Descriptive data	14*	(a) Give characteristics of study participants (eg demographic, clinical, social) and information on exposures and potential confounders	Table 1
		(b) Indicate number of participants with missing data for each variable of interest	Appendix
		(c) Summarise follow-up time (eg, average and total amount)	N/A
Outcome data	15*	Report numbers of outcome events or summary measures over time	N/A

Main results	16	(*a*) Give unadjusted estimates and, if applicable, confounder-adjusted estimates and their precision (eg, 95% confidence interval). Make clear which confounders were adjusted for and why they were included	5-7
		(*b*) Report category boundaries when continuous variables were categorized	5-7
		(*c*) If relevant, consider translating estimates of relative risk into absolute risk for a meaningful time period	N/A
Other analyses	17	Report other analyses done—eg analyses of subgroups and interactions, and sensitivity analyses	Appendix
**Discussion**			
Key results	18	Summarise key results with reference to study objectives	3
Limitations	19	Discuss limitations of the study, taking into account sources of potential bias or imprecision. Discuss both direction and magnitude of any potential bias	9
Interpretation	20	Give a cautious overall interpretation of results considering objectives, limitations, multiplicity of analyses, results from similar studies, and other relevant evidence	8-9
Generalisability	21	Discuss the generalisability (external validity) of the study results	8-9
**Other information**			
Funding	22	Give the source of funding and the role of the funders for the present study and, if applicable, for the original study on which the present article is based	5
